# Development and validation of a nomogram for predicting deep venous thrombosis in patients with pelvic and acetabular fractures: a retrospective cohort study: Predictive model for pelvic/acetabular fractures

**DOI:** 10.1186/s12891-023-06879-9

**Published:** 2023-10-02

**Authors:** Zongyou Yang, Ren Rongqing, Zhizhou Yang, Hucheng Yang, Yingchao Yin, Siyu Tian, Zhihong Wang, Zhiyong Hou

**Affiliations:** 1 Department of Orthopaedic Surgery, Third Hospital of Hebei Medical University, No. 139 Zi Qiang Road, Shijiazhuang, 050051 Hebei P.R. China; 2 Orthopaedic Research Institute of Hebei Province, Shijiazhuang, Hebei China; 3 Key Laboratory of Intelligent Orthopaedic Equipment, National Health Commission (NHC), The Third Hospital of Hebei Medical University, Shijiazhuang, Hebei China

**Keywords:** Venous thrombosis, Pelvis, Acetabula, Fractures, Risk factors, Nomograms

## Abstract

**Purpose:**

To construct a novel nomogram model that can predict DVT and avoid unnecessary examination.

**Methods:**

Patients admitted to the hospital with pelvis/acetabular fractures were included between July 2014 and July 2018. The potential predictors associated with DVT were analyzed using Univariate and multivariable logistic regression analysis. The predictive nomogram was constructed and internally validated.

**Results:**

230 patients were finally enrolled. There were 149 individuals in the non-DVT group and 81 in the DVT group. Following analysis, we obtained the final nomogram model. The risk factors included age (OR, 1.037; 95% CI, 1.013–1.062; P = 0.002), body mass index (BMI) (OR, 1.253; 95% CI, 1.120–1.403; P < 0.001); instant application of anticoagulant after admission (IAA) (OR, 2.734; 95% CI, 0.847–8.829; P = 0.093), hemoglobin (HGB) (OR, 0.970; 95% CI, 0.954–0.986; P < 0.001), D-Dimer(OR, 1.154; 95% CI, 1.016–1.310; P = 0.027) and fibrinogen (FIB) (OR, 1.286; 95% CI, 1.024–1.616; P = 0.002). The apparent C-statistic was 0.811, and the adjusted C-statistic was 0.777 after internal validations, demonstrating good discrimination. Hosmer and Lemeshow’s goodness of fit (GOF) test of the predictive model showed a good calibration for the probability of prediction and observation (χ^2^ = 3.285, P = 0.915; P > 0.05). The decision curve analysis (DCA) and Clinical impact plot (CIC) demonstrated superior clinical use of the nomogram.

**Conclusions:**

An easy-to-calculate nomogram model for predicting DVT in patients with pelvic-acetabular fractures were developed. It could help clinicians to reduce DVT and avoid unnecessary examinations.

## Introduction

Surgeons encounter considerable difficulties when managing pelvic and acetabular fractures, primarily due to the elevated incidence of complications, including male sexual dysfunction, limb dysfunction [1, 2]. Occasionally, these fractures can prove to be fatal, particularly when combined with preoperative deep venous thrombosis (DVT). It is reported that the incidence of a thromboembolic event is 13.5-33.7% and even 57.6% in severe traumatic cases [3–6]. To monitor and identify DVT, colour Doppler ultrasound is applied frequently. Consequently, changing body position during examination imposes extensive pain on patients sustaining pelvic or acetabular fractures. Meanwhile, the fixed legs of patients also make it difficult for physicians to get clear and accurate results. Therefore, a predictive model to identify the patients suffering the high risk and predict DVT early might help clinicians to take prophylactic measures to reduce the incidence of DVT and avoid unnecessary examination. Notably, the nomogram model is a novel and convenient tool and has been widely used to predict outcomes and complications in various diseases [7–11]. To date, several investigations have evaluated the risk factors of venous thromboembolism [3–5, 12, 13]. However, none of them developed a nomogram. Hence, we designed the retrospective cohort study and aimed to construct and validate a novel nomogram model for predicting DVT in individuals with pelvic and acetabular fractures.

## Methods

### Data sources and participants

This protocol has been approved by the ethical board (G2020-029-1). From July 2014 to July 2018, all the in-hospital individuals diagnosed with pelvis/acetabular fractures were recorded. The inclusion criteria were as follows: (1) adult patients (older than eighteen years old), (2) those diagnosed with pelvic/acetabular fractures, and (3) the complete medical data was available. Exclusion criteria are as follows: (1) patients with other fractures, (2) open fractures or non-traumatic fractures, (3) received multiple surgeries, (4) present with haematological diseases or the current use of anticoagulant drugs, and (5) patients were admitted later than fourteen days since injury.

### Data collection

All the patients enrolled underwent basic examinations, including a blood test and electrocardiogram check after admission. X-rays or computed tomography (CT) of the pelvis were performed as soon as the patients’ situations were stable. Those who were unsuitable for surgery were treated conservatively. Additionally, the patients were scheduled for operating procedures as soon as they met the indications for surgery. At our institution, two sonographers consistently employed color Doppler ultrasound to routinely detect deep vein thrombosis (DVT) in the bilateral lower extremities and pelvis, starting from admission and continuing up to one week post-surgery. Based on the review of previous investigations and experts’ suggestions in traumatology, we screened 23 predictors. The clinical data of patients with pelvic-acetabular fractures were retrieved from the picture archiving and communication system (PACS) and electronic medical record system (EMRS). The general information included age and gender. The lifestyle and anthropometric factors included body mass index (BMI) and smoking history. The injury-associated factors included injury mechanism, time from injury to admission, combined injury, and the American Society of Anesthesiologists (ASA) classification. The injury caused by bicycle accidents or fall from a standing height was classified as low-energy injury, and the injury caused by motor vehicle traffic accidents or falls from high places was classified as high-energy injury. The comorbidities included diabetes mellitus, hypertension, and visceral injury. The treatment factors included the instant application of anticoagulant after admission (IAA), a history of anticoagulant use, timing of surgery and surgery techniques. The patients were treated with those techniques such as percutaneous screw fixation (PSF), external fixation (EF) and open reduction and internal fixation (ORIF). The haematological predictors included white blood cell (WBC), red blood cell (RBC), platelet (PLT), hemoglobin (HGB), triglyceride (TG), random blood glucose (RBG), D-Dimer level, prothrombin time (PT), activated partial thromboplastin time (APTT), international normalized ratio (INR) and fibrinogen (FIB).

### Statistical analysis

Categorical variables were assessed using the Chi-square test. These categorical variables were revealed as percentages. The statistical differences of continuous variables were compared by a two-sample-t test or Mann-Whitney U test, and they were presented as means ± standard deviation (SD). The odds ratio (OR) with a 95% confidence interval (CI) was also calculated. The predictors with bilateral p < 0.20 in the univariate were admitted to the multivariable logistic regression analysis in the backward stepwise elimination method. The factors with p-values greater than or equal to 0.05 were discarded. Along with the multivariable logistic regression analysis, a nomogram predicting the probability of deep venous thrombosis in patients with pelvic-acetabular fractures was constructed using the “rms” package. Notably, we accessed the predictive model’s performance with calibration and discrimination. Calibration plots and the Hosmer-Lemeshow goodness-of-fit (GOF) tests were applied to access calibration. Discrimination was evaluated with the concordance statistic index (c-index) or the area under the curve value (AUC). Furthermore, all the analyses were conducted with IBM SPSS Statistics (version 25.1, IBM Corp, Armonk, NY, USA) or R software (version 4.2.0, R Foundation for Statistical Computing, Vienna, Austria, http://www.R-project.org).

## Results

### Demographic and clinical characteristics

**Fig. 1:**
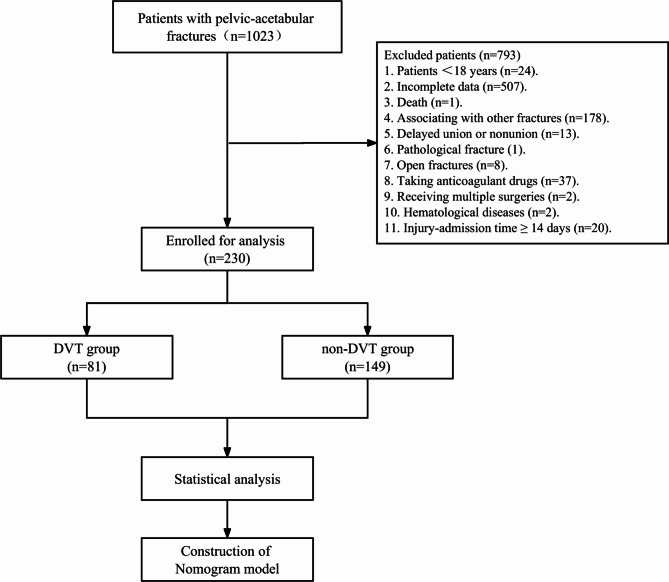
The workflow of the patients with pelvic-acetabular fractures enrollment

From January 2014 to August 2018, 1,023 patients with pelvic-acetabular fractures were admitted to the Third Hospital of Hebei Medical University. Of all, 24 patients were under eighteen years old. Five hundred-seven cases were excluded due to incomplete data. One patient could not accomplish the whole treatment due to sudden death during hospitalization. One hundred seventy-eight patients were associated with femoral, tibial, and/or other type of fractures, which closely affected the development of deep venous thrombosis. Thirteen patients were diagnosed as delayed union or nonunion. One individual had cancer with pelvis metastasis. Eight patients suffered from open fractures. Thirty-seven patients were on the duration of taking aspirin, clopidogrel, and/or other anticoagulant drugs. 2 cases received debridement after the surgery. 2 patients had haematological diseases. Twenty patients were admitted later than fourteen days after injury. A total of 230 patients were finally enrolled (Fig. 1). Based on the colour Doppler ultrasound results, the patients enrolled were divided into the non-DVT group (n = 149) and the DVT group (n = 81). The baseline levels of the characteristics of patients with pelvic-acetabular fractures are listed in Table 1.

**Table 1:** Demographic and clinical characteristics of patients with pelvic/acetabular fractures. IAA, instant application of anticoagulant after admission

Factor	non-DVT group (n = 149)	DVT group (n = 81)	P
Age, years (mean ± SD)	42.95 ± 14.28	53.04 ± 14.54	< 0.001
Gender, n (%)			0.833
Female	44(29.53%)	25(30.86%)	
Male	105(70.47%)	56(69.14%)	
BMI, Kg/m2 (mean ± SD)	23.75 ± 2.94	25.38 ± 2.74	< 0.001
History of smoking, n (%)			0.149
No	124(83.22%)	61(75.31%)	
Yes	25(16.78%)	20(24.69%)	
Mechanism, n (%)			0.752
low	63(42.28%)	36(44.44%)	
high	86(57.72%)	45(55.56%)	
Injury-admission time, days (mean ± SD)	0.87 ± 1.86	1.94 ± 3.14	0.008
Comorbidities, n (%)			0.546
Diabetes	1	1	
Hypertension	7	2	
Visceral injury	14	10	
ASA score, n (%)			0.009
1	26(17.45%)	4(4.94%)	
2	93(62.42%)	51(62.96%)	
3	30(20.13%)	26(32.10%)	
IAA, n (%)			0.018
No	23(15.44%)	4(4.94%)	
Yes	126(84.56%)	7(95.06%)	
History use of anticoagulant, n (%)			0.471
No	145(97.32%)	80(98.77%)	
Yes	4(2.68%)	1(1.23%)	
Timing of surgery	152.13 ± 80.63	159.40 ± 67.74	0.209
Surgery technique			0.848
PSF	47	25	
EF	10	4	
ORIF	92	52	
WBC, x109/L (mean ± SD)	10.31 ± 3.81	10.11 ± 3.84	0.713
RBC, x1012/L (mean ± SD)	4.00 ± 0.74	3.56 ± 0.64	< 0.001
PLT, x109/L (mean ± SD)	210.63 ± 74.27	202.67 ± 81.55	0.453
HGB, g/L (mean ± SD)	116.33 ± 23.15	102.08 ± 18.77	< 0.001
TG, mmol/L (mean ± SD)	1.10 ± 0.62	1.22 ± 0.64	0.160
RBG, mmol/L (mean ± SD)	6.39 ± 1.94	6.83 ± 1.97	0.107
D-Dimer, mg/L (mean ± SD)	2.15 ± 2.15	3.38 ± 3.51	0.003
PT, s (mean ± SD)	12.23 ± 1.22	12.20 ± 1.05	0.837
APTT, s (mean ± SD)	29.49 ± 3.67	29.27 ± 4.08	0.683
INR, (mean ± SD)	1.10 ± 0.11	1.10 ± 0.10	0.877
FIB, g/L (mean ± SD)	3.37 ± 1.31	3.85 ± 1.64	0.019

### Risk factors screening

There were 105 males and 44 females in the non-DVT group and 56 males and 25 females in the DVT group, which showed no significant difference. The patients in the DVT group were statistically older and were admitted to the hospital one more days later on average than those in the non-DVT group (P < 0.001 and P = 0.008, respectively). The high-energy injury was the most common mechanism injury in both groups. The BMI was higher in the DVT group than in the non-DVT group (P < 0.001). There were 25 (16.78%) patients who had a history of smoking in the non-DVT group and 20 (24.69%) patients in the DVT group (P = 0.1485). There was no significant difference between the two groups in the history of anticoagulant drug use and comorbidities. In addition, the ASA score was statistically different between the two groups (P = 0.009). While the timing of surgery appeared to have no marked influence on the forming of DVT (P = 0.209). The results of surgery techniques exhibited no difference compared to their counterparts between the two groups (P = 0.848). Through t-test analysis, we found a significant association of DVT with several blood parameters: RBC, HGB, D-Dimer, and FIB (P < 0.05). Additionally, the P value in TG and FBG were 0.160 and 0.107, respectively. All the variables with a P value less than 0.20 were identified as candidate predictors, including age, BMI, smoking history, injury-admission time, ASA score, IAA, RBC, HGB, TG, RBG, D-Dimer, and FIB. Then those candidate predictors were entered into the multivariate logistic regression analysis.

Six predictors remaining in the final model were suggested to be independent risks for predicting DVT in the patients with pelvic-acetabular fractures: age (OR, 1.037; 95% CI, 1.013–1.062; P = 0.002), BMI(OR, 1.253; 95% CI, 1.120–1.403; P < 0.001); IAA (OR, 2.734; 95% CI, 0.847–8.829; P = 0.093), HGB (OR, 0.970; 95% CI, 0.954–0.986; P < 0.001), D-Dimer(OR, 1.154; 95% CI, 1.016–1.310; P = 0.027) and FIB (OR, 1.286; 95% CI, 1.024–1.616; P = 0.002; Table 2).

**Table 2:** Results of multivariate Logistic regression analysis of DVT in patients with pelvic-acetabular fractures. IAA, instant application of anticoagulant after admission

Factor	β	S.E.	Wald	Crude odds ratio	95% CI	P-value
Age	0.037	0.012	9.474	1.037	(1.013,1.062)	0.002
BMI	0.226	0.058	15.404	1.253	(1.120,1.403)	< 0.001
IAA	1.006	0.598	2.830	2.734	(0.847,8.829)	0.093
HGB	-0.031	0.008	13.844	0.970	(0.954,0.986)	< 0.001
D-Dimer	0.143	0.065	4.874	1.154	(1.016,1.310)	0.027
FIB	0.252	0.116	4.671	1.286	(1.024,1.616)	0.031
Constent	-7.770	2.085	13.885	-	-	-

### Nomogram development and internal validation

**Fig. 2:**
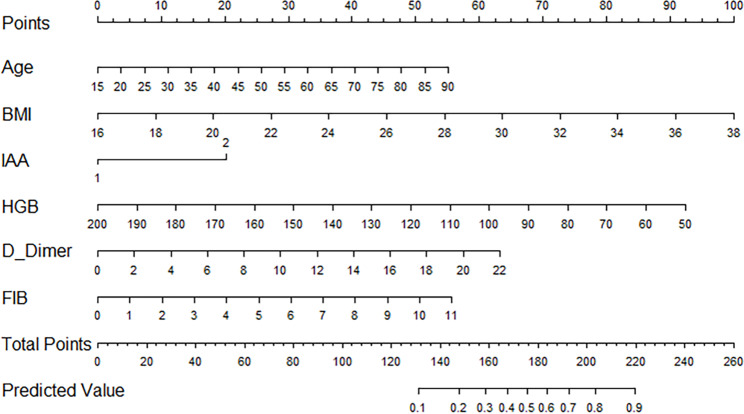
Nomogram to predict the risk of deep venous thrombosis in patients with pelvic-acetabular fractures and its predictive performance

**Fig. 3:**
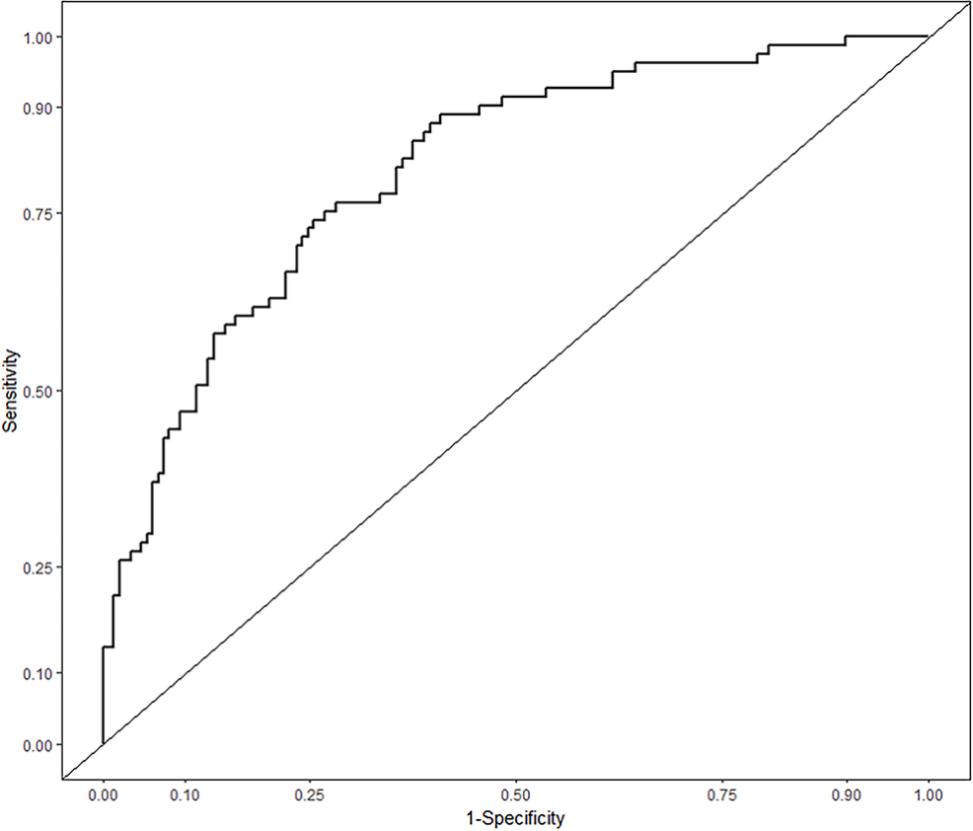
Receiver operating characteristic (ROC) curves for the prediction of deep venous thrombosis in patients with pelvic-acetabular fractures

**Fig. 4:**
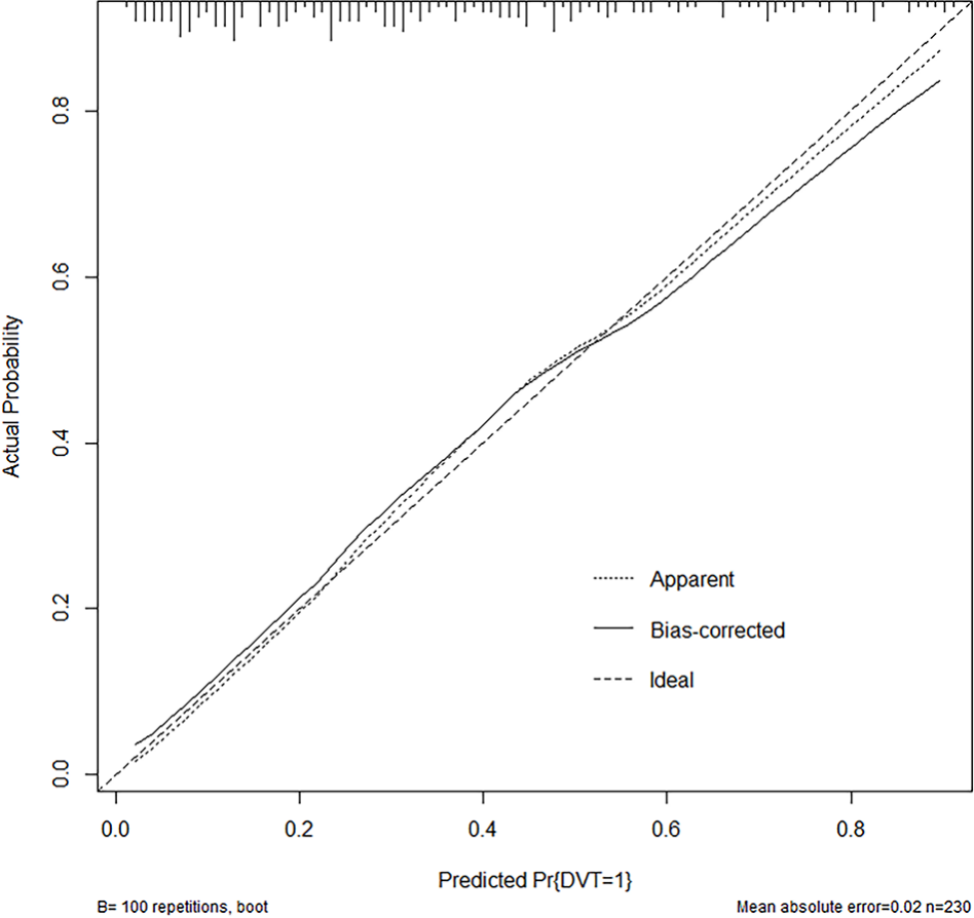
Calibration curves of the nomogram. The dashed line represents the original performance, and the solid dashed line represents the performance during internal validation by bootstrapping (B = 100 repetitions)

All six aforementioned predictors were incorporated in the development of a nomogram aimed at predicting the likelihood of deep venous thrombosis in patients with pelvic-acetabular fractures, spanning from admission to one week after surgical intervention. (Fig. 2). The apparent C-statistic estimated the model discrimination as per the six predictors with a value of 0.811 (Fig. 3). The adjusted C-statistic was 0.777 after 100 bootstrapping internal validations, demonstrating good discrimination. In addition, the calibration plot showed an excellent fit during internal validation (Fig. 4). GOF test of the predictive model showed a good calibration for the probability of prediction and observation (χ^2^ = 3.285, P = 0.915; P > 0.05). The Brier score was 0.165, and the adjusted brier score was 0.176.

### Clinical practice

**Fig. 5:**
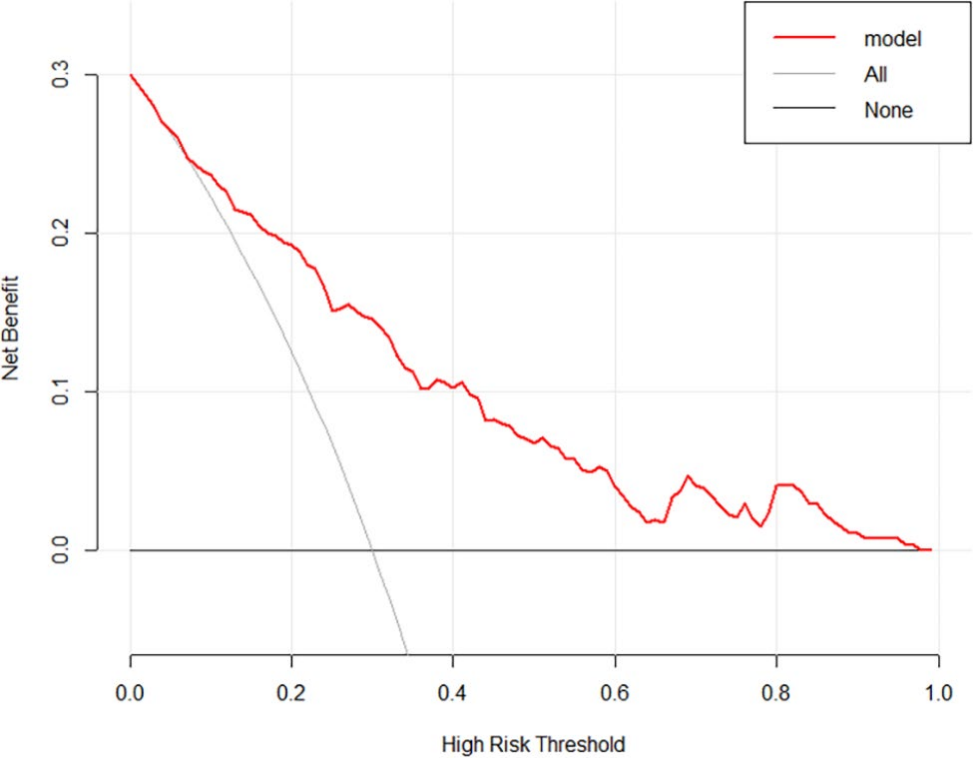
Decision curve analysis of the nomogram

**Fig. 6:**
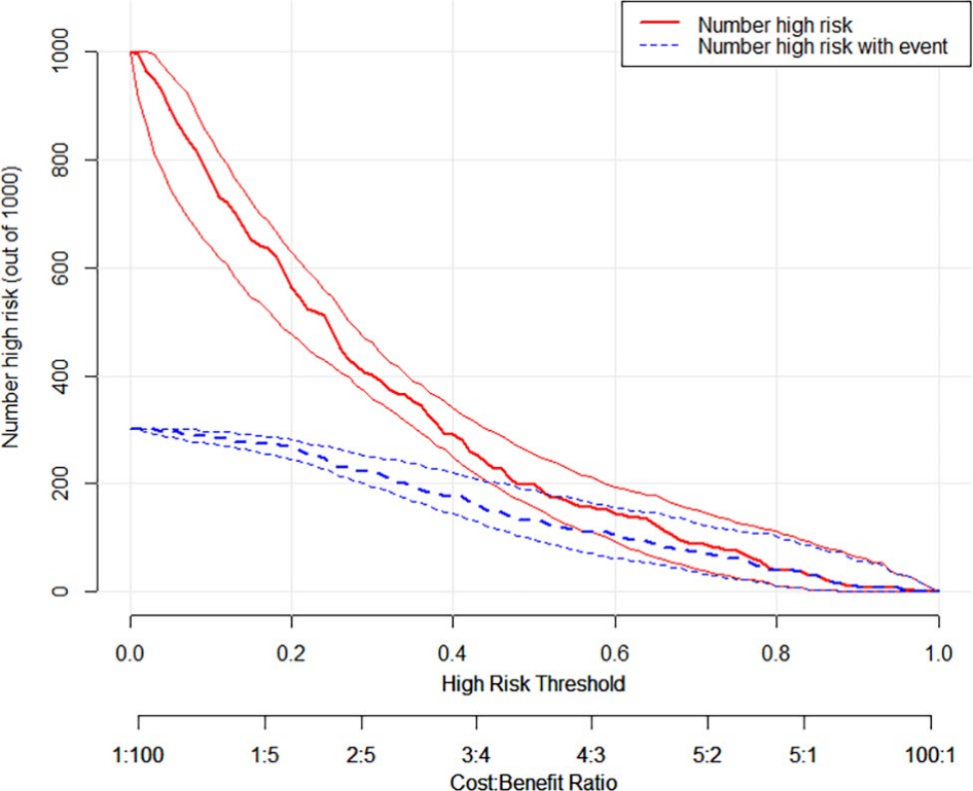
Clinical impact curve detects the predictive value of the nomogram

The decision curve analysis (DCA) of the predictive model was performed (Fig. 5). The vertical axis measures the net benefit, and the horizontal measures the high-risk threshold (Threshold Probability). As shown in the figure, for an extensive range of threshold probability (from 0.1 to 0.9), the treatment decision based on the predictive model leads to higher benefits than the schemes of examining all patients regularly or examining none patients. A clinical impact plot (CIC) was subsequently produced (Fig. 6). Of 1,000 patients, the solid line represents the total number of patients who would be declared high risk for each risk threshold. The dashed line represents the number of true-positive patients. The CIC results demonstrated that the number of cases deemed positive based on the nomogram is greater than the number of true positives. Furthermore, with the risk threshold increasing, the false positives decline gradually.

## Discussion

DVT is a common complication in traumatic fractures, especially in pelvic-acetabular fractures [14]. Although the physicians in our hospital are experienced and pay much attention to avoiding DVT in patients with pelvic-acetabular fractures, the incidence of DVT in our study was as high as 35.2% (81/230). With increased awareness, some physicians use routine colour Doppler ultrasound measures to monitor venous thrombosis. However, the examination is painful for patients and makes it difficult for examiners to diagnose accurately. Notably, several investigations have evaluated the risk factors to reduce the incidence rates of venous thromboembolism [3–5, 12, 13]. Two of the studies were based on the case-control design [3, 4] and one on cross-sectional design [13]. Another study by Wand [12] included lower extremities injury, which directly affected developing DVT and inevitably increased the confounding bias. Kim and colleagues [5] had not considered haematological indicators, which play key roles in blood coagulation, and reduced the reliability of results. To our knowledge, this is the first study to establish a nomogram model to predict individualized risk for DVT in patients with pelvic-acetabular fractures.

Here, we identified 6 independent risk factors for DVT: age, BMI, IAA, HGB, D-Dimer, and FIB. Age is a high potential risk factor for developing venous thrombosis in trauma fractures, which is widely accepted. We observed that age was a significant factor affecting DVT. The mean age of the group with DVT is 10.09 years older than this without DVT (53.04 years vs. 42.95 years). The multivariate Logistic regression analysis result suggests that patients with higher age are associated with a higher risk for DVT (OR = 1.037). The results of Yan et al. [4] support our observations, despite some minute differences in the odds ratio (OR = 1.070). Some other studies focused on the mechanism which underlies the association. The study by Culmer et al. [15] demonstrates that adhesion molecules, such as P-selectin, are important. The increasing expression of P-selectin with age contributes to a pro-thrombotic environment and facilitates thrombus initiation. Another etiology study by Andrea et al. [16] reveals that the age-related metabolites glutamine, phenylalanine, and proline significantly affect the vein wall and P-selectin, contributing to venous thrombosis. However, the mechanism is not well understood. The relationship between age and deep venous thrombosis in patients with pelvic-acetabular fractures needs further research and exploration.

Body mass index (BMI) is an established factor for many kinds of diseases [17–20], including venous thrombosis [21]. The overweight is defined as a BMI of 25-29.9 kg/m^2^. When the BMI is no less than 30 kg/m^2^, it is considered obese. Notably, obesity is regarded as a significant risk for venous thrombosis. The correlation between BMI and thrombosis is strengthened with the increment in BMI. The odds ratios of obesity are reported at a range of 1.7–3.9 [3, 22, 23]. Our study found that every 1 kg/m^2^ increase in BMI resulted in 1.253-time odds ratios to developing DVT in patients with pelvic-acetabular fractures. Notwithstanding the controversy of the underlying mechanism, BMI is still an independent predictor for DVT.

The prevalence of DVT is much higher in patients with pelvic-acetabular fractures compared to non-trauma patients. The application of pharmacological anticoagulation is the critical prophylaxis option for DVT. Even so, the definitive DVT prophylaxis is controversial, and thromboprophylaxis should be initialed as soon as patients are no longer at risk for hemorrhea [24, 25]. 133 out of 230 individuals received IAA after admission. The Chi-square test shows a statistical difference between the groups that receive IAA or not (P = 0.018). However, after adjusting the confounding factors by multivariable logistic regression analysis, the difference was not as significant as before (P > 0.05).

Fibrinogen, the most abundant circulating coagulation protein, is synthesized and secreted by the liver. D-Dimer is the common biochemical indicator for venous thrombosis. As shown in Table 1, significant differences in FIB and D-Dimer are found between the non-DVT and DVT groups. The increase in the FIB and D-Dimer levels contributes to the increased risk for DVT in trauma patients with pelvic-acetabular fractures (OR = 1.286; OR = 1.154). Interestingly, we found that those patients suffering from anemia were prone to DVT. Furthermore, Zhang et al. [26] reported that preoperative anemia was closely related to DVT in trauma patients, similar to our findings.

There is currently no other nomogram specifically designed to predict DVT in patients with pelvic-acetabular fractures. Importantly, we have identified 6 high-risk factors and established a quantitative and practical nomogram for clinicians. In the internal validation, our nomogram demonstrated satisfying discrimination and calibration. The predictors involved are well-defined and convenient to collect. Meanwhile, it is easy to calculate total points and predictive value accuracy. From decision curve analysis, our nomogram showed a good net benefit gain, suggesting that it has a superior clinical potential to influence reasonable clinical decisions and improve clinical outcomes.

However, this study has some shortages. Firstly, we conducted a single-center study. Although internal validation was performed, there is still no external validation. Secondly, we included only Chinese patients. Thus, external validation must be done to confirm that it applies to other populations of patients with pelvic-acetabular fractures.

## Conclusion

The nomogram for predicting DVT in patients with pelvic-acetabular fractures, based on age, BMI, IAA, HGB, D-Dimer, and FIB, was an easy-to-calculate model. It has excellent potential to reduce DVT and avoid unnecessary examinations.

## Data Availability

The data used to support the findings of this study are available from the corresponding author upon request.

## Abbreviations

DVT: Deep venous thrombosis
CT: Computed tomography
PACS: The picture archiving and communication system
EMRS: Electronic medical record system
BMI: Body mass index
ASA: American Society of Anesthesiologists
IAA: Anticoagulant after admission
PSF: Percutaneous screw fixation
EF: External fixation
ORIF: Open reduction and internal fixation
WBC: White blood cell
RBC: Red blood cell
PLT: Platelet
HGB: Hemoglobin
TG: Triglyceride
RBG: Random blood glucose
PT: Prothrombin time
APTT: Activated partial thromboplastin time
INR: International normalized ratio
FIB: Fibrinogen
SD: Means ± standard deviation
OR: Odds ratio
CI: Confidence interval
AIC: Akaike information criterion
GOF: Goodness-of-fit
AUC: The area under the curve
DCA: Decision curve analysis
CIC: Clinical impact plot
ROC: Receiver operating characteristic
